# The effect of postal questionnaire burden on response rate and answer patterns following admission to intensive care: a randomised controlled trial

**DOI:** 10.1186/s12874-017-0319-3

**Published:** 2017-03-27

**Authors:** Robert Hatch, Duncan Young, Vicki Barber, David A Harrison, Peter Watkinson

**Affiliations:** 1 Kadoorie Centre for Critical Care Research and Education, University of Oxford, John Radcliffe Hospital, Headley Way, Oxford, OX3 9DU UK; 2 Registrar in Anaesthetics, Oxford Deanery, Oxford, UK; 3 Intensive Care Medicine, University of Oxford, Oxford, UK; 4 OCTRU Hub, University of Oxford, Oxford, UK; 5 Statistician, Intensive Care National Audit and Research Centre (ICNARC), London, UK; 6 University of Oxford and Consultant Intensive Care Physician, Oxford University Hospitals NHS Trust, Oxford, UK

**Keywords:** Health related quality of life, EQ5D, Questionnaire, Response rate, Randomised control trial, Intensive care, Critical care, Survivors, Multicentre study, Critical illness, Outcome assessment

## Abstract

**Background:**

The effects of postal questionnaire burden on return rates and answers given are unclear following treatment on an intensive care unit (ICU). We aimed to establish the effects of different postal questionnaire burdens on return rates and answers given.

**Methods:**

Design: A parallel group randomised controlled trial. We assigned patients by computer-based randomisation to one of two questionnaire packs (Group A and Group B).

Setting: Patients from 26 ICUs in the United Kingdom.

Inclusion criteria: Patients who had received at least 24 h of level 3 care and were 16 years of age or older. Patients did not know that there were different questionnaire burdens. The study included 18,490 patients. 12,170 were eligible to be sent a questionnaire pack at 3 months. We sent 12,105 questionnaires (6112 to group A and 5993 to group B).

Interventions: The Group A pack contained demographic and EuroQol group 5 Dimensions 3 level (EQ-5D-3 L) questionnaires, making four questionnaire pages. The Group B pack also contained the Hospital Anxiety and Depression Score (HADS) and the Post-Traumatic Stress Disorder Check List-Civilian (PCL-C) questionnaires, making eight questionnaire pages in total.

Main outcome measure: Questionnaire return rate 3 months after ICU discharge by group.

**Results:**

In group A, 2466/6112 (40.3%) participants responded at 3 months. In group B 2315/ 5993 (38.6%) participants responded (difference 1.7% CI for difference 0–3.5% *p* = 0.053).

Group A reported better functionality than group B in the EQ-5D-3 L mobility (41% versus 37% reporting no problems *p* = 0.003) and anxiety/depression (59% versus 55% reporting no problems *p* = 0.017) domains.

**Conclusions:**

In survivors of intensive care, questionnaire burden had no effect on return rates. However, questionnaire burden affected answers to the same questionnaire (EQ-5D-3 L).

**Trial registration:**

ISRCTN69112866 (assigned 02/05/2006).

**Electronic supplementary material:**

The online version of this article (doi:10.1186/s12874-017-0319-3) contains supplementary material, which is available to authorized users.

## Background

Self-completed postal questionnaires provide a convenient, cost-effective method of measuring patient outcomes, avoiding travel for participants and researchers. However, non-participation has been increasing over time [1]. If non-participation is not random, the sampled population may differ from the whole population and may affect the generalisability and usefulness of the results [2].

Many researchers have investigated methods of improving responses to postal questionnaires. Their findings have been extensively reviewed [3–6]. Varied methods such as using a package of communication strategies, teasers on envelopes, personalising the questionnaire, non-monetary incentives and making clear that the study is based at a university have all been found to improve response rates [5]. Questionnaire burden may affect participation in self-completed postal questionnaires [5, 6]. It may also alter the answers given [7].

Changes in quality of life, mental and physical health occur after treatment on an Intensive Care Unit (ICU) [8–12]. In the Intensive Care Outcome Network (ICON) study we investigated quality of life, mental and physical health following treatment on an ICU using validated postal questionnaires. Whether questionnaire burden affects return rates or answers from patients after treatment on an ICU is unknown. In other hospitalised patient populations, we found two randomised studies, with conflicting results [7, 13].

We therefore undertook an early example of a Study Within a Trial (SWAT) [14] to investigate the effects of questionnaire burden on participation and answers.

## Methods

### Study design

We conducted a randomised controlled trial as a study within a trial (SWAT), within the ICON study. The ICON study assessed quality of life, the incidence of depression, and the incidence of post-traumatic stress disorder following at least 24 h treatment on an ICU; the protocol has been published [15]. The study received national ethics approval (REC 06/Q1605/17) and local research governance approval was obtained at each centre.

### Study population

Patients from 26 UK ICUs took part (1 university hospital, 6 university-affiliated hospitals and 19 district general hospitals). We gave all patients a letter introducing the study at ICU discharge: it explained that they might receive mail from the study team. Patients were eligible if they received level 3 care (as defined by the Intensive Care Society, London [16]) on an ICU for at least 24 h. We excluded patients if they were under 16 years old. We also excluded patients not registered with a general practitioner or of no fixed abode (factors anticipated to prevent follow-up in the study). We excluded patients taking part in another questionnaire follow-up study run by the same research office.

### Allocation

We used a pseudo-random number generator, built into GNU Libc (http://www.gnu.org/software/libc/), to allocate patients to a study group without restriction. Allocation occurred at the central research office at the point of enrolment.

### Interventions

The group A pack contained four questionnaire pages. The pack contained the one page EuroQol 5 dimensions 3 level (EQ-5D-3 L) questionnaire, two pages each containing a single visual analogue scale and a single page demographics questionnaire.

The group B pack contained eight questionnaire pages. In addition to the group A pack, it also contained a two page Hospital Anxiety and Depression Scale (HADS) questionnaire and a two page Post-traumatic stress disorder Check List– Civilian version (PCL-C) questionnaire.

The initial packs sent at 3 months contained a personally addressed letter inviting participation. The 3-month packs also contained a three-page study information leaflet and a consent form (required by the Ethics Committee).

Packs at 12 and 24 months contained a covering letter and the questionnaires, but no information leaflet or consent form. However, if patients did not receive a 3-month pack because they remained in hospital, the 12-month pack contained the initial letter, study information leaflet and consent form. Questionnaire content remained the same at 3, 12 and 24 months.

Participants were unaware that we were sending different questionnaire packs.

### Survey implementation

Both groups received questionnaire packs 3 months after ICU discharge. We sent further questionnaire packs at 12 and 24 months after ICU discharge to respondents who agreed to take part further. We sent repeat questionnaire packs if a reply did not arrive after 2 weeks. Repeat questionnaire packs were identical to the first pack other than slight changes to the wording of the letter that made it clear that this was a repeat mailing. We did not contact patients who spent over 75 days in hospital following their discharge from ICU at 3 months, instead these patients were first contacted at 12 months. Patients who remained in hospital at 12 months did not receive any follow up.

We checked survival with the patient’s registered general practitioner and the National Health Service clinical spine application before posting each questionnaire pack. If the general practitioner informed us that a participant would be unable to complete a questionnaire, we did not send a pack.

We printed all documents using a high quality laser printer. We printed the invitation letter on Oxford University headed paper. The trial coordinator signed each letter. We printed each questionnaire on different coloured paper and bound them with a removable clip. All pages were single-sided and numbered. We used uniform design, large font size and generous spacing. All packs contained a Freepost addressed envelope for questionnaire return. All packs included an ICON branded pen with an ICON-labelled tea bag as incentives; the tea bag label invited the participant to enjoy a cup of tea whilst completing the questionnaire. Participants could also complete the questionnaires by telephone with a trained researcher.

### Outcome measures

The primary outcome measure was the questionnaire return rate 3 months after ICU discharge, by group. For the first mailed pack we defined questionnaire return as a completed consent form agreeing to take part in the study and at least one questionnaire question or visual analogue scale completed and returned. For subsequent packs we defined questionnaire return as at least one questionnaire question or visual analogue scale completed and returned.

Secondary outcome measures were return rates at 12 and 24 months and the effect of questionnaire burden on the EQ-5D-3 L weighted index score and individual domain scores at 3 months after ICU discharge. We defined a valid response as completion of all questions within an instrument. We undertook a post-hoc analysis of missingness on the invalid returns.

### Sample size

The ICON study ran for 19 months before the randomised controlled trial started. During this time we sent 6028 patients the group B pack and a Short Form 36 version 2 (SF36v2—health related quality of life questionnaire) at 3 months. The 3 month questionnaire return rate was 36%. We assumed a similar return rate for group B packs sent in the trial. We estimated 18,000 patients would take part in the next 28 months (allowing for site changes). Assuming a similar mortality 75 days following discharge from ICU (32%), power of 90% and a significance level of 0.05, this sample size was sufficient to detect a 2.9% change in the return rate.

### Statistical analysis

We used an electronic form reader (Teleform v10, Cambridge, UK) to transcribe questionnaire responses into a database (MySQL v5.0-Oracle Corporation, Redwood Shores, CA). Study office personnel manually entered data that the electronic form reader could not interpret. We linked participant records with the Intensive Care National Audit & Research Centre (ICNARC) Case Mix Programme database to obtain admitting diagnoses and severity of illness scores [17].

Statistical analysis was undertaken using R Core v3.2.3 [18]. We used Fisher’s exact test to compare between group rates of questionnaire return. We analysed questionnaire return rates for all patients to whom we sent a questionnaire. We used the Mann–Whitney “U” test to compare EQ-5D-3 L weighted index scores and visual analogue scales between groups at the first time point. We used the Chi-squared test to compare the proportion of patients who responded as EQ-5D-3 L level 1 (“No problems”) with a single collapsed category for those who responded as level 2 (“moderate”) or level 3 (“severe”) [19]. We did not correct for multiple testing. We did not include information from EQ-5D-3 L respondents with any missing or invalid domain responses in our analysis of responses to the EQ-5D-3 L questionnaire.

## Results

**Fig. 1:**
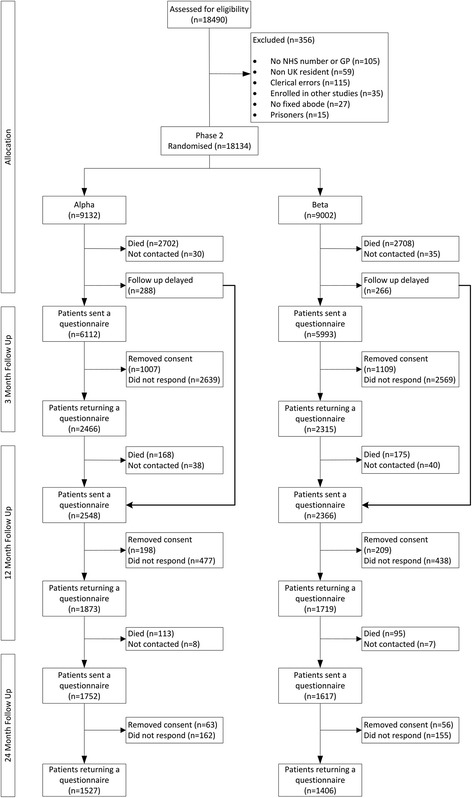
Consort diagram

Patients joined the study May 2008-September 2010 inclusive as planned. The study database closed 28 months after recruitment of the final patient. Of the 18,490 patients who were screened, 18,134 underwent randomization (Fig. 1). Table 1 shows the characteristics of randomised patients by group. Table 2 is a response analysis showing the characteristics of those patients that responded to the study at 3 months (an equivalent non-response analysis is included in Additional file 1: Table S1). Table 3 shows response rates at 3, 12 and 24 months by group. Response rates were equivalent at all time points.

**Table 1:** Demographics

	Group A (*n* = 9132)	Group B (*n* = 9002)	All (*n* = 18,134)
Age median, [IQR]	66 [52–76]	67 [53–76]	66 [52–76]
Male sex (%)	56%	56%	56%
APACHE II score median, [IQR]	16 [12–21]	16 [12–21]	16 [12–21]
ICU length of stay days median [IQR]	3 [2–7]	3 [2–6]	3 [2–7]
Hospital length of stay days median [IQR]	14 [7–27]	14 [7–28]	14 [7–28]
Reason for ICU admission *n* (%)			
Respiratory tract infection	966 (11)	972 (11)	1938 (11)
Major vascular procedure	453 (5)	428 (5)	881 (5)
Large bowel tumour	383 (4)	454 (5)	837 (5)
Acute renal failure	439 (5)	420 (5)	859 (5)
Chronic obstructive pulmonary disease	283 (3)	292 (3)	575 (3)
Bowel perforation	278 (3)	226 (3)	504 (3)
Septicaemia/septic shock	304 (3)	300 (3)	604 (3)
Oesophageal neoplasm	130 (1)	136 (2)	266 (1)
Status epilepticus	198 (2)	174 (2)	372 (2)
Self-poisoning	290 (3)	291 (3)	581 (3)
Not recorded	301 (3)	309 (3)	610 (3)
Other	5107 (56)	5000 (56)	10,107 (56)

*n* (%), median [interquartile range]

**Table 2:** Responders at 3 months

		Group A (*n* = 2466)	Group B (*n* = 2315)	*p*	All (*n* = 4781)
Age median, [IQR]		65 [53–74]	65 [53–74]	0.99^c^	65 [53–74]
Male sex (%)		57%	57%	0.87^b^	57%
APACHE II score median, [IQR]		15 [11–19]	14 [11–19]	0.08^a^	15 [11–19]
ICU length of stay days median [IQR]		3 [2–6]	3 [2–6]	0.33^a^	3 [2–6]
Hospital length of stay days median [IQR]		15 [9–26]	15 [8–25]	0.23^a^	15 [8–25]
Reason for ICU admission *n* (%)					
Respiratory tract infection		237 (10)	192 (8)	0.16^b^	429 (9)
Major vascular procedure		185 (8)	171 (7)	0.93^b^	356 (7)
Large bowel tumour		148 (6)	173 (7)	0.07^b^	321 (7)
Acute renal failure		109 (4)	90 (4)	0.42^b^	199 (4)
Chronic obstructive pulmonary disease		79 (3)	76 (3)	0.95^b^	155 (3)
Bowel perforation		66 (3)	47 (2)	0.18^b^	113 (2)
Septicaemia/septic shock		60 (2)	61 (3)	0.73^b^	121 (3)
Oesophageal neoplasm		56 (2)	70 (3)	0.14^b^	126 (3)
Status epilepticus		52 (2)	44 (2)	0.69^b^	96 (2)
Self-poisoning		44 (2)	53 (2)	0.27^b^	97 (2)
Not recorded		77 (3)	64 (3)	0.53^b^	141 (3)
Other		1353 (55)	1274 (55)		2627 (55)
Self-reported demographics *n* (%)					
Current address	Usual address	2305 (93)	2165 (94)		4470 (93)
	Family	36 (1)	38 (2)		74 (2)
	Rehabilitation	19 (1)	11 (0)		30 (1)
	Other	36 (1)	38 (2)		74 (2)
	Not usual (unspecified)	13 (1)	10 (0)		23 (0)
	Missing	57 (2)	53 (2)	0.79^b^	110 (2)
Higher education	Yes	985 (40)	983 (42)		1968 (41)
	No	1235 (50)	1138 (49)		2373 (50)
	Missing	246 (10)	194 (8)	0.07^b^	440 (9)
University degree	Yes	580 (24)	600 (26)		1180 (25)
	No	1700 (69)	1579 (68)		3279 (69)
	Missing	186 (8)	136 (6)	0.02^b^	322 (7)
Current employment status	Full time	245 (10)	213 (9)		458 (10)
	Part time	108 (4)	129 (6)		237 (5)
	Seeking	27 (1)	35 (2)		62 (1)
	Retired	1353 (55)	1250 (54)		2603 (54)
	Sick	380 (15)	376 (16)		756 (16)
	Housework	120 (5)	110 (5)		230 (5)
	Student	24 (1)	17 (1)		41 (1)
	Other	112 (5)	97 (4)		209 (4)
	Missing	97 (4)	88 (4)	0.47^b^	185 (4)
Prior employment status	Full time	437 (18)	388 (17)		825 (17)
	Part time	189 (8)	190 (8)		379 (8)
	Seeking	38 (2)	24 (1)		62 (1)
	Retired	1233 (50)	1170 (51)		2403 (50)
	Sick	168 (7)	169 (7)		337 (7)
	Housework	132 (5)	105 (5)		237 (5)
	Student	27 (1)	16 (1)		43 (1)
	Other	75 (3)	87 (4)		162 (3)
	Missing	167 (7)	166 (7)	0.28^b^	333 (7)
Carer now	Yes	150 (6)	152 (7)		302 (6)
	No	2207 (89)	2052 (89)		4259 (89)
	Missing	109 (4)	111 (5)	0.64^b^	220 (5)
Carer prior	Yes	159 (6)	170 (7)		329 (7)
	No	1844 (75)	1680 (73)		3524 (74)
	Missing	463 (19)	465 (20)	0.2^b^	928 (19)
Assistance	Answered	246 (10)	288 (12)		534 (11)
	Unable	68 (3)	42 (2)		110 (2)
	Unspecified	21 (1)	26 (1)		47 (1)
	No	2050 (83)	1893 (82)		3943 (82)
	Missing	81 (3)	66 (3)	0.01^b^	147 (3)
Mental problems now	Yes	334 (14)	298 (13)		632 (13)
	No	2034 (82)	1912 (83)		3946 (83)
	Missing	98 (4)	105 (5)	0.52^b^	203 (4)
Mental problems prior	Yes	354 (14)	331 (14)		685 (14)
	No	1730 (70)	1636 (71)		3366 (70)
	Missing	382 (15)	348 (15)	0.9^b^	730 (15)

*n* (%), median [interquartile range]

*p* values not corrected for multiple testing

^a^Mann-Whitney *U* test (non-parametric)

^b^Chi-squared test

^c^Welch’s *t*-test

**Table 3:** Three, 12 and 24 month response rates

	3 months		12 months		24 months	
	Group A	Group B	Group A	Group B	Group A	Group B
Posted (*n*)	6112	5993	2548	2366	1752	1617
Response (*n*)	2466	2315	1873	1719	1527	1406
Response Rate (%)	40.3	38.6	73.5	72.7	87.2	87.0
Absolute difference (%)	1.7		0.9		0.2	
95% CI	0.0 to 3.5		−1.7 to 3.4		−2.1 to 2.5	
Relative Risk	1.03		1.03		1.02	
95% CI	1.00 to 1.06		0.94 to 1.13		0.85 to 1.21	
*p*	0.053		0.52		0.878	

We randomised 18,134 of the 18,490 patients assessed for inclusion (see Fig. 1). 5410 patients died within 3 months of ICU discharge. We delayed follow-up until 12 months in 554 patients who spent over 75 days in hospital. 12,170 patients were eligible to receive a questionnaire pack at 3 months. Table 1 shows demographic data for all participants. Table 2 is a response analysis showing the characteristics of those patients that responded to the study at 3 months (an equivalent non-response analysis is included in Additional file 1: Table S1). Between the two groups responders were very similar, although, without correction for multiple testing, differences existed in self-reported university education and need for assistance. Table 3 shows response rates at 3, 12 and 24 months by group. Response rates were equivalent at all time points.

Table 4 shows valid responses to the EQ-5D-3 L questionnaire at 3 months by group. Patients in group B reported worse function in the “anxiety and depression” (*p* = 0.017) and “mobility” (*p* = 0.003) domains of the EQ-5D-3 L questionnaire at 3 months. Questionnaire burden did not affect answers to the “activities”, “pain/discomfort” and “self-care” domains at 3 months. Nor did it affect median EQ-5D-3 L weighted index scores or EQ visual analogue scale score at 3 months. Participants did not always complete every dimension. An analysis of missingness is shown in Additional file 2: Table S2.

**Table 4:** Three month questionnaire responses

	Group A		Group B		
EuroQol 5 Dimension 3 Level (EQ-5D-3 L)					
Valid Responses	*n* = 2399 (97)		*n* = 2263 (98)		
Mobility					
level 1	978	(41)	826	(37)	*p* = 0.003^a^
level 2	1382	(58)	1407	(62)	
level 3	39	(2)	30	(1)	
Self Care					
level 1	1656	(69)	1572	(69)	*p* = 0.771^a^
level 2	687	(29)	648	(29)	
level 3	56	(2)	43	(2)	
Usual Activities					
level 1	716	(30)	626	(28)	*p* = 0.107^a^
level 2	1323	(55)	1254	(55)	
level 3	360	(15)	383	(17)	
Pain/Discomfort					
level 1	785	(33)	742	(33)	*p* = 0.986^a^
level 2	1386	(58)	1318	(58)	
level 3	228	(10)	203	(9)	
Anxiety/Depression					
level 1	1409	(59)	1250	(55)	*p* = 0.017^a^
level 2	836	(35)	864	(38)	
level 3	154	(6)	149	(7)	
EQ-5D-3 L weighted index score	0.69	[0.52–0.81]	0.69	[0.52–0.81]	*p* = 0.255
EuroQol Visual Analogue Scale (EQ VAS)					
Valid Responses	*n* = 2402 (97)		*n* = 2244 (97)		
EQ VAS Score	70	[50–80]	66	[50–80]	*p* = 0.174

*n* (%), median [interquartile range]

^a^Chi-square test comparing level 1 “No problems” with a single collapsed category combining level 2-“moderate” and level 3-“severe”

## Discussion

Halving the questionnaire burden had no effect on response rates. Most participants who returned a questionnaire at 3 months completed questionnaires at later follow-up points. The group sent the long questionnaire pack reported worse function in the “mobility” and “anxiety and depression” EQ-5D-3 L domains.

We believe our study is by far the largest randomised controlled trial of questionnaire burden. Our short questionnaire pack was the EQ-5D-3 L (four pages). The EQ-5D-3 L was the shortest validated quality of life questionnaire for patients recovering from critical illnesses. Our long questionnaire pack also contained HADS and PCL-C questionnaires (an extra four pages).

We believe we followed best practice for postal questionnaires [3, 5, 6, 20]. Packs included an ICON branded pen and teabag as non-monetary incentives. We used a non-financial incentive appropriate to the situation as these have evidence of benefit and because a financial incentive would have been too costly given the scale of our study [5, 21]. We used personalised letters headed on University of Oxford paper that detailed the patients name, address and their admission hospital [3, 5]. We printed each questionnaire on different coloured paper bound with a removable clip [5]. We used a package of postal communications, including sending out 2 copies of the questionnaires [3]. We used good data management practices to optimise our data capture. This included using only single-sided pages to ensure participants did not miss questions printed on the back of pages and numbering the pages [5]. We used a large font size and generous spacing to facilitate responses from older patients [22].

Our study has limitations. We only contacted patients by post, limiting our findings to postal-only questionnaires. We stopped sending questionnaires to patients who did not respond at the previous mailing point, however recovery from critical illness may have increased responses over time. Additional telephone contact may have changed our results, although this took place very rarely. Our short questionnaire pack contained nine pages. Five of these pages were the letter, consent form and information leaflet. These pages were necessary as patients did not agree to take part before leaving hospital. Informed consent before discharge would have been a more complex approach. However, a (shorter) pack without these pages may have improved return rates [5].

Two studies have examined the effect of questionnaire burden in patients discharged from hospital [7, 13]. In the International Stroke trial more participants responded to a six question EuroQol instrument than the longer SF-36 questionnaire [7]. Yet, this difference was not seen with Picker Patient Experience questionnaires of different lengths [13].

Three systematic reviews have assessed the effect of reducing questionnaire burden on return rates [3, 5, 6]. They all included participants who had not recently been in hospital. Two report an increase in return rates with decreased questionnaire burden [5, 6]. The most recent, restricted to clinical randomised controlled trials found only a marginal effect [3]. In patients recovering from severe illness, return rates to postal questionnaires are variable [7–12].

In the International Stroke Trial [7], where return rates were higher than in our study, patients agreed to take part in the trial before they received questionnaires. In the ICON study, we combined agreeing to take part and returning the first questionnaire. This combination may explain our lower initial return rates. Where patients agreed to take part in our study, later return rates were similarly high. In our study the longer questionnaire group reported worse function in the “mobility” and “usual activities” EQ-5D-3 L domains.

Two trials in recently hospitalised patients also studied the relationship between questionnaire burden and the answers given. Different Picker Patient Experience questionnaire lengths did not affect the answers given [13]. Conversely, more stroke survivors scored themselves as dependent using the (shorter) six question EuroQol instrument than the SF-36 instrument [7]. The difference seen may reflect the different questionnaires used, rather than the questionnaire burden. In our study, questionnaire burden affected the answers given to the EQ-5D-3 L questionnaire. When presented with additional questionnaires four percent of respondents placed themselves in a worse category.

In patients discharged from an ICU, our study is large enough to be definitive. Reducing questionnaire length from eight to four pages has little effect on return rates. However, including extra questionnaires resulted in different EQ-5D-3 L answers. This interaction suggests investigators should avoid mailings with multiple questionnaires. The effect adds to the case for minimising the burden to participants.

We undertook this study before the Study Within a Trial (SWAT) programme commenced [14, 23]. Our study demonstrates that large scale SWATs examining clinical outcomes can be undertaken. Our findings highlight the potential impact of trial methodologies on outcomes.

With the same response rate in the two groups, we did not expect different answers to EQ-5D-3 L domains. The reasons underlying the different answers remain unclear. The extra questions may have resulted in a different response group. Alternatively, the extra questions may have caused the same group to respond differently. We need to understand better the links between questionnaire burden and the pattern of answers.

## Conclusions

In patients treated on an intensive care unit questionnaire burden affected the findings from the same questionnaire. This is a compelling reason to minimise the questionnaire burden. Halving the number of questionnaire pages had no effect on the return rate.

## Additional files

Additional file 1: Table S1. Non-response Analysis. (DOCX 14 kb)

Additional file 2: Table S2. Missingness Analysis. (DOCX 12 kb)

## Abbreviations

EQ-5D-3 L: EuroQol group 5 Dimensions 3 level
HADS: Hospital Anxiety and Depression Score
ICNARC: Intensive Care National Audit & Research Centre
ICON: Intensive Care Outcome Network
ICU: Intensive Care Unit
PCL-C: Post-Traumatic Stress Disorder Check List-Civilian
REC: Research Ethics Committee
SF36v2: Short Form 36 version 2
SWAT: Study Within a Trial
UK: United Kingdom
