# Supplementary material for: The effect of postal questionnaire burden on response rate and answer patterns following admission to intensive care: a randomised controlled trial
Source: BMC Med Res Methodol. 2017 Mar 27;17:49. doi: 10.1186/s12874-017-0319-3 (PMC5368992; doi:10.1186/s12874-017-0319-3)
Supplement: Supplementary file 1 — Non-response Analysis. (DOCX 14 kb) [file 12874_2017_319_MOESM1_ESM.docx]

Appendix Table 1. Non-response Analysis

|  | **Group A (n=3646)** | **Group B (n=3678)** | **p** | **All (n= 7324)** |
| --- | --- | --- | --- | --- |
| Age median, [IQR] | 62 [ 44 - 74 ] | 63 [ 45 - 74 ] | 0.01 # | 62 [ 44 - 74 ] |
| Male sex (%) | 56 | 55 | 0.91 * | 55 |
| APACHE II score median, [IQR] | 14 [ 11 - 19 ] | 14 [ 11 - 19 ] | 0.79 # | 14 [ 11 - 19 ] |
| ICU length of stay days median [IQR] | 3 [ 2 - 6 ] | 3 [ 2 - 6 ] | 0.32 # | 3 [ 2 - 6 ] |
| Hospital length of stay days median [IQR] | 14 [ 7 - 27 ] | 15 [ 8 - 29 ] | 0.04 # | 14 [ 7 - 28 ] |
|  |  |  |  |  |
| **Reason for ICU admission n (%)** |  |  |  |  |
| Respiratory tract infection | 274 (8) | 343 (9) | 0.01 ~ | 617 (8) |
| Major vascular procedure | 175 (5) | 178 (5) | 0.98 ~ | 353 (5) |
| Large bowel tumour | 153 (4) | 196 (5) | 0.03 ~ | 349 (5) |
| Acute renal failure | 168 5) | 145 (4) | 0.20 ~ | 313 (4) |
| Chronic obstructive pulmonary disease | 102 (3) | 113 (3) | 0.54 ~ | 215 (3) |
| Bowel perforation | 100 (3) | 83 (2) | 0.22 ~ | 183 (2) |
| Septicaemia/septic shock | 90 (2) | 106 (3) | 0.32 ~ | 196 (3) |
| Oesophageal neoplasm | 56 (2) | 55 (1) | 0.96 ~ | 111 (2) |
| Status epilepticus | 99 (3) | 100 (3) | 1.00 ~ | 199 (3) |
| Self-poisoning | 226 (6) | 211 (6) | 0.46 ~ | 437 (6) |
| Not recorded | 121 (3) | 149 (4) | 0.12 ~ | 270 (4) |
| Other | 2082 (57) | 1999 (54) |  | 4081 (56) |

n (%), median [interquartile range].

p values not corrected for multiple testing

# Mann–Whitney U test (non-parametric)

~ Chi-squared test

* Welch's t-test
