# Supplementary material for: The effect of postal questionnaire burden on response rate and answer patterns following admission to intensive care: a randomised controlled trial
Source: BMC Med Res Methodol. 2017 Mar 27;17:49. doi: 10.1186/s12874-017-0319-3 (PMC5368992; doi:10.1186/s12874-017-0319-3)
Supplement: Supplementary file 2 — Missingness Analysis. (DOCX 12 kb) [file 12874_2017_319_MOESM2_ESM.docx]

Appendix Table 2. Missingness Analysis

|  | **Group A (n=67)** | **Group B (n=52)** |
| --- | --- | --- |
| **Number of domains missing per patient response** |  |  |
| 1 | 49 (73.1) | 35 (67.3) |
| 2 | 7 (10.4) | 4 (7.7) |
| 3 | 3 (4.5) | 0 (0) |
| 4 | 2 (3.0) | 0 (0) |
| 5 | 6 (9.0) | 13 (25.0) |
|  |  |  |
| **Number of missing responses per domain** |  |  |
| 1 | 25 | 25 |
| 2 | 21 | 21 |
| 3 | 21 | 16 |
| 4 | 20 | 22 |
| 5 | 23 | 23 |

n (%)
